# On and Under the Skin: Emerging Basidiomycetous Yeast Infections Caused by *Trichosporon* Species

**DOI:** 10.1371/journal.ppat.1004982

**Published:** 2015-07-30

**Authors:** Marçal Mariné, Neil Andrew Brown, Diego Mauricio Riaño-Pachón, Gustavo Henrique Goldman

**Affiliations:** 1 Faculdade de Ciências Farmacêuticas de Ribeirão Preto, Universidade de São Paulo, São Paulo, Brazil; 2 Laboratório Nacional de Ciência e Tecnologia do Bioetanol—CTBE, Campinas, São Paulo, Brazil; Duke University Medical Center, UNITED STATES

## What Are *Trichosporon* Species?

**Fig 1:**
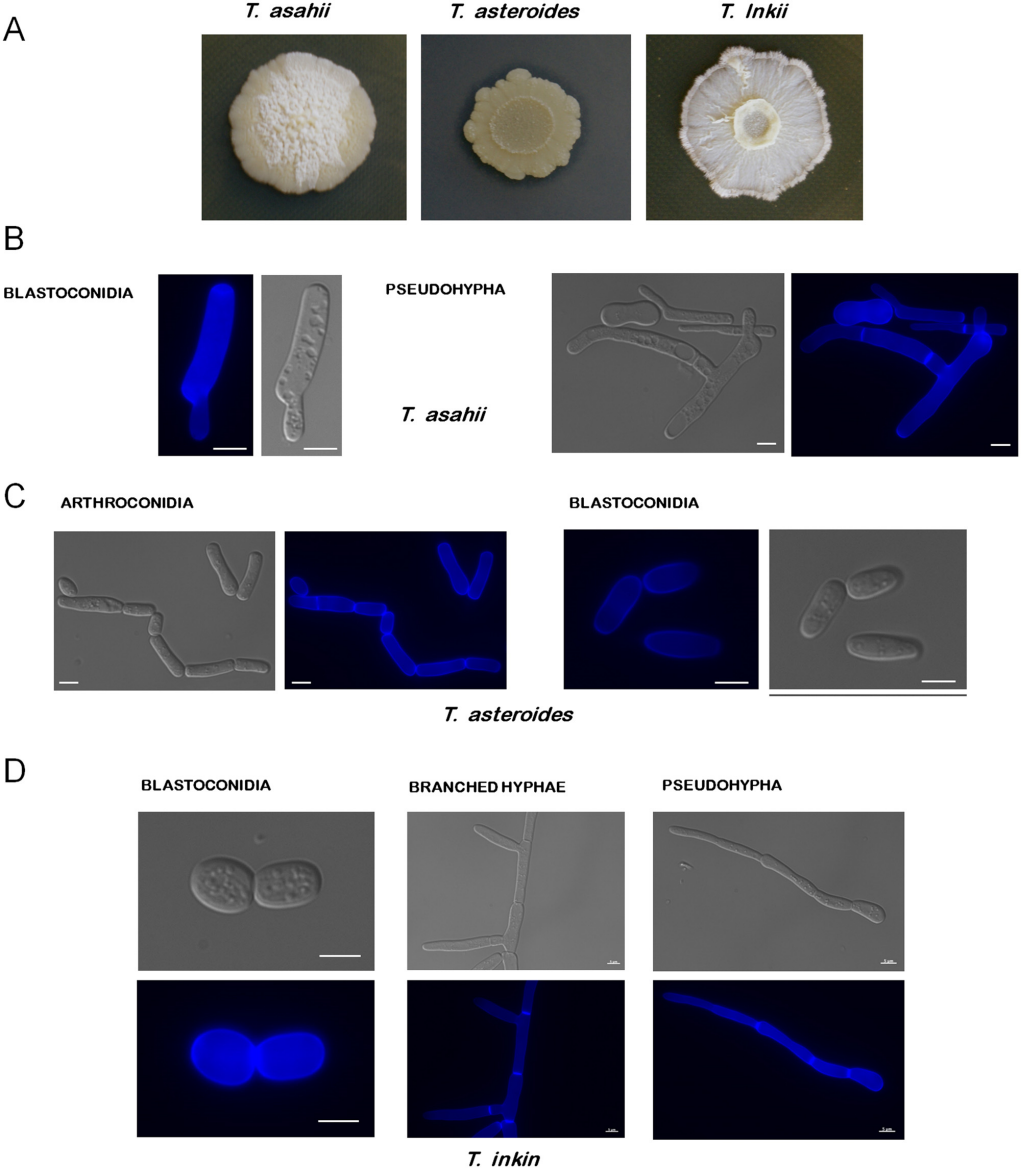
Colony morphology of *Trichosporon* species. (A) *T*. *asahii*, *T*. *asteroides*, and *T*. *inkii* were grown at 30°C for 15 days on Sabouraud, YNB+tributyrin 1%, and YPD media, respectively. *T*. *asteroides* is releasing lipases, producing a halo where tributyrin is degraded. (B) *T*. *asahii* blastoconidia and pseudohypha. (C) *T*. *asteroides* arthroconidia and blastoconidia. (D) *T*. *inkin* pseudohypha, branched hypha, and blastoconidia. The blue fluorescence on the structures is due to their incubation with calcofluor white. Bars, 5 μm.

*Trichosporon* species are basidiomycetous, yeast-like organisms capable of filamentous growth, i.e., dimorphic (Fig 1), that are distributed throughout nature [1]. They are important from a biotechnological point of view as they are capable of decontaminating polluted environments by accumulating large amounts of oils [2–5]. A limited number of reports also show their presence in the human microbiome, such as *Trichosporon asahii*, which has been isolated from human fecal samples, the skin of healthy individuals, and patients with atopic dermatitis [1,6–10]. In addition, *Trichosporon* spp. can cause white piedra, hypersensitivity pneumonitis, superficial infections, and invasive trichosporonosis [11].

**Fig 2:**
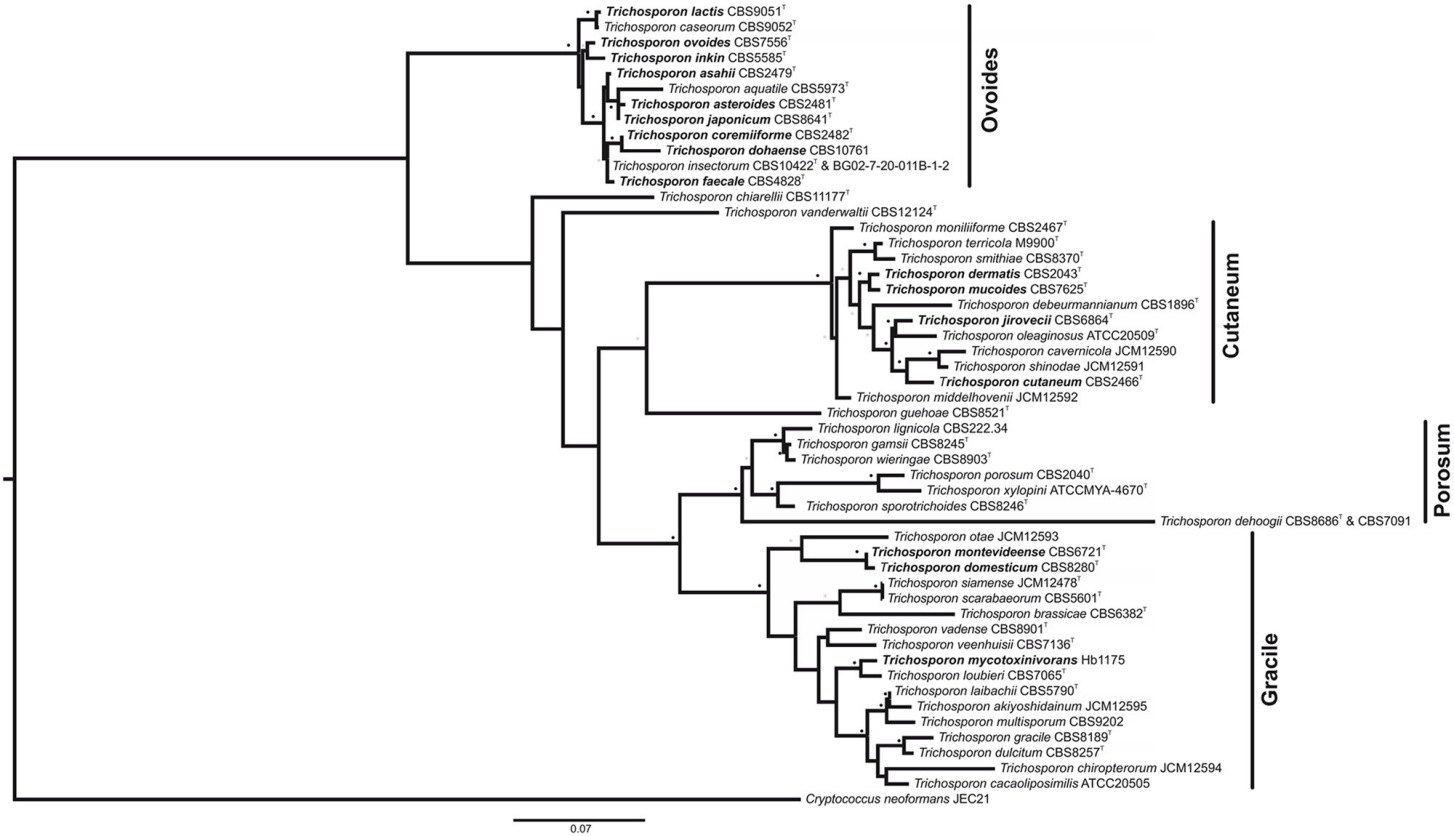
Maximum-likelihood phylogenetic tree of *Trichosporon* species, based on analysis of the ITS1 and ITS2 regions and the D1/D2 region of the LSU. Strain names appear after the species name. *Trichosporon* species of clinical significance appear in bold. See S1 Table for the GenBank accession numbers. *Cryptococcus neoformans* was used as an outgroup. Sequences of each individual region were structurally aligned using MXSCARNA [58] and then concatenated into a supermatrix using FASConCAT [59]. Phylogenetic inference was carried out with the software RAxML [60]. For the loop regions, the evolutionary model GTR was used. The stem regions were analyzed under the S16 evolutionary model, thus taking into account secondary structure topology, i.e., compensatory mutations [61–63]. Substitution rate heterogeneity was taken into account using the gamma model of Yang [64]. Bootstrap values were computed over 1,000 replicates. Grey dots represent bootstrap values 50%–75% and black dot bootstrap values 76%–100%. Bar, 0.07 substitutions per nucleotide position. T: Type strain.

Designated 150 years ago, the genus *Trichosporon* was for many years a collection of many different yeast-like organisms. Until the end of the 20th century, a wide range of species were included under the name of *Trichosporon beigelii* or synonyms, which were later shown to be phylogenetically distinct. With the advent of molecular techniques, the genus was rearranged, and many of its species were reassigned to other genera and new ones were described. Currently there are 51 accepted *Trichosporon* species, 16 of which have clinical relevance [1,12–13] (these species are highlighted in Fig 2).

Formerly associated with uncommon hair and skin infections, research on *Trichosporon* has fallen behind more life-threatening fungi such as *Candida* or *Cryptococcus* species, until the rise of opportunistic, deeply seated, disseminated *Trichosporon* infections (especially *T*. *asahii*) over the last decades. A lack of background knowledge impairs the proper diagnosis and treatment of *Trichosporon* infections. Fortunately in the recent years, studies of *Trichosporon* epidemiology [14], virulence factors [15,16], antifungal susceptibility [16,17], and animal models of infection [18,19] have been conducted. There are a few reports of genetic transformation systems for *T*. *cutaneum*, which used both dominant and auxotrophic markers [20,21]. However, forward and reverse genetics is still not common practice in the study of *Trichosporon*.

Molecular techniques have already proved necessary for the detection and correct identification of *Trichosporon* species [1,17]. However, the use of whole genome sequencing and gene manipulation techniques in *Trichosporon* is in its infancy, in comparison to other fungi, such as *Candida* or *Aspergillus* species. Genome sequences for a *T*. *asahii* environmental strain and a strain isolated from a progressive psoriatic lesion are available [22,23]. Both *T*. *asahii* genomes are of approximately 25 Mbp and are predicted to encode approximately 9,000 genes. A comparison of these two *Trichosporon* genomes revealed >99% chromosomal and mitochondrial sequence identity. When compared to the genomes of other skin-associated or pathogenic basidiomycetes, the *T*. *asahii* genome is larger and is predicted to encode a greater number of genes than the basidiomycetes *Malassezia restricta*, *M*. *globosa*, and *M*. *sympodialis*, which range from 7.6 to 9.0 Mbp and are predicted to encode around 3,500 to 4,300 genes [24,25]. In contrast, the genomes of *Cryptococcus neoformans* and *C*. *gattii* are between 17 to 20 Mbp and are predicted to encode around 6,500 to 8,300 genes [26,27].

Along with the advances in health care, rarer opportunistic pathogens are gaining more attention, and through the anticipated application of modern pathogenomics to the study of trichosporonosis, this may represent a turning point in the understanding of *Trichosporon* species and their virulence determinants.

## What Is the Clinical Importance of *Trichosporon*?

Skin and hair infections due to *Trichosporon* were considered rare for a long time, but the misdiagnosis of superficial trichosporonosis might have led to an underestimation of its prevalence [28]. Being a part of the natural microbiota of the skin, these *Trichosporon* species can be misdiagnosed as contaminants, resulting in the infection being attributed to dermatophytes, when in fact *Trichosporon* might be the etiological agent in 10%–40% of superficial infections depending on the geographic area and population [29,30]. The old designation *T*. *beigelii* has been used years after the rearrangement of the genus [29]. The carryover of older names is common in clinical practice and can interfere in the correct diagnosis and treatment. In order to overcome this issue, fast and efficient molecular-based tools are being developed to effectively identify *Trichosporon* species in clinical settings [31]. In addition, MALDI-TOF mass spectrometry has proven to be a reliable tool for the identification of *Trichosporon* species and could become a cheaper and complementary alternative to gene sequencing [32,33]. Currently *T*. *inkin*, *T*. *cutaneum*, *T*. *ovoides*, and *T*. *loubieri* are considered the most prominent species involved in superficial trichosporonosis, while *T*. *asahii*, *T*. *ateroides*, and *T*. *mucoides* are associated with invasive infections in immunocompromised patients [11]. *T*. *asahii* stands out as the leading cause of disseminated infections and is not usually associated with superficial infections [2,34]. However, a rare case was recently reported of a *T*. *asahii* cutaneous infection that progressed into subcutaneous tissues and resulted in a fatal outcome in an immunocompetent patient [35].

The vast majority of cases of disseminated trichosporonosis occur in patients that are immunocompromised or those that have received cytotoxic chemotherapy, steroids, or broad-spectrum antibiotics [14,36]. A major risk factor is reported to be the use of venous catheters or drains that may facilitate the penetration of the fungus beyond the colonized skin [37]. In a global surveillance study, *Trichosporon* species accounted for a 0.5% of total yeast isolates and 10.7% of non-*Candida* species in invasive and mucosa-associated infections [38]. Invasive *Trichosporon* infections have been reported in 0.4% of patients with acute leukemia and are associated with a high mortality rate of 64% [14]. This is also a common trend for hematological patients, since high mortality rates, ranging from 42% to 76%, have been widely reported for *Trichosporon* infections [39]. Apart from cancer and neutropenia, other underlying conditions, such as burns, surgery, and organ failure, were reported to be associated with invasive *Trichosporon* infections and a mortality rate as high as 87.5% in adult patients in Brazil [34].

## How Can *Trichosporon* spp. Infections Be Treated?

Superficial *Trichosporon* infections, such as white piedra, often respond well to topical or oral azole treatments combined with improved hygienic habits to avoid relapses [40]. However, invasive infections represent a therapeutic challenge, and no consensus exists for a recommended treatment. Several studies report minimal success with amphotericin B or fluconazole [11,37], which correlates with the commonly reported high minimal inhibitory concentrations for these drugs [34,36]. The echinocandins display minimal activity against basidiomycetes, and several cases of breakthrough infections caused by *Trichosporon* on patients undergoing echinocandin therapy have been reported [41,42]. Only a single case has been reported of the successful treatment of peritonitis caused by *T*. *inkin* with caspofungin alone [43]. Newer azole drugs such as voriconazole and posaconazole have shown excellent in vitro activity against *Trichosporon* [16,17]. In vivo efficacy and increasing reports of successful treatments with voriconazole highlight this drug as a potential therapeutic option to combat disseminated *Trichosporon* infections [11,44,45]. Posaconazole, on the other hand, has also shown encouraging results in a murine model of infection [19] but lacks clinical evidence of its efficacy. Combined antifungal therapy is considered a way to broaden the antimycotic spectrum of treatments, especially applicable to cases of rare or refractory fungal infections. However, the use of multiple antifungal agents can lead to antagonistic interactions and cause interference with other therapies the patient might be receiving. Hence, this option is only used as a last resort, unless there is an important patient background that encourages its use [46]. Concomitant or sequential usage of antifungal drugs has been reported for *Trichosporon* infections and generally yielded undistinguished results [14,37]. However, experimental results and recent reports of successful treatments [18,47] suggest that combined therapies are an option to take into account against trichosporonosis.

## How Do *Trichosporon* spp. Colonize and Infect the Hosts?

With the exception of *C*. *neoformans*, basidiomycete disseminated infections are rare and tend to occur in patients with severe underlying conditions. *Cryptococcus* disseminated infections are generally associated with a primary pulmonary infection that disseminates through the bloodstream and tends to affect the central nervous system [48]. Several filamentous basidiomycetes, such as *Hormographiella aspergillata* and *Tyromyces fissilis* [49,50], have also been reported to cause pulmonary infections in severely immunocompromised patients, supposedly by spore inhalation. Conversely, several basidiomycetous yeasts, such as *Trichosporon*, *Rhodotorula*, *Malassezia*, and *Sporobolomyces* species, are considered to colonize or even infect the skin or mucosa, a location from which they take advantage of a barrier disruption caused by their own means or by trauma, such as a catheter implantation.

The ability of *Trichosporon* to invade the skin and other tissues requires several pathogenic traits, such as biofilm formation; enzymatic activities, including phospholipases and proteases; and the production of hyphae or pseudohyphae. Both *Trichosporon* and *Malassezia* are good examples of host-adapted pathogens that possess, to some extent, all the aforementioned traits. Biofilm formation has been observed in several *Trichosporon* species [16], a trait that is closely related to the ability to grow on exogenous surfaces such as a catheter. Central venous catheter removal is strongly recommended in cases of blood-positive cultures of *Trichosporon*, *Malasezzia*, and *Rhodotorula* in the same manner as it is recommended in bloodstream infections by *Candida* [11,51,52]. *C*. *neoformans* has a wide array of pathogenic traits that permit it to avoid the host immune system and to cross natural barriers within the body; however, not much is known about its ability to form biofilms, which is proposed to be lower than that of the other pathogenic yeasts mentioned above [8]. Accordingly, clinical reports about *C*. *neoformans* association to a central venous catheter are scarce [53]. One of the most well-known virulence traits of *Cryptococcus* species is the production of the polysaccharide glucuronoxylomannan that protects the cells from phagocytosis, collaborates in evasion of the immune system, and promotes intracellular survival [49]. *Trichosporon* species also produce glucuronoxylomannan, which if demonstrated to have a role in virulence, would represent a possible therapeutic target [54].

## How Do *Trichosporon* spp. Adapt to the Host?

The production of enzymes capable of degrading various components of host tissues is also a major virulence trait present in many pathogenic fungi [11]. A recent study demonstrated that several species of *Trichosporon* are capable of producing a variety of proteases, lipases phospholipases, and DNAases [15]. This hydrolytic activity is a well-known trait that allows other basidiomycetous yeasts, such as *Malassezia* and *Rhodotorula*, to obtain fatty acids from host lipids and breakdown phospholipids present in cell membranes. The proteolytic activity of *Trichosporon* is a trait shared with dermatophytes that produce a plethora of proteases, which in accordance with their ecological niche, play an important role in overcoming host immunological barriers [25].

In contrast to *Trichosporon* species, dermatophytes and *Malassezia* have undergone a severe loss of genes encoding for carbohydrate-degrading enzymes, which indicates a high degree of adaptation to animal hosts [25]. The animal skin is the common target of dermathophytes and *Malassezia*; however, they adopt different approaches to pathogenesis. *Malassezia* predominately obtain nourishment from host fatty acids, to the point that they have lost the metabolic routes to synthesize them [25]. Dermatophytes, on the other hand, are specialized in the degradation of keratin and other proteins [55]. These ascomycetes also present a wide arsenal of immunomodulators, antimicrobial and toxic compounds, as well as many of secondary metabolites of unknown function that reveal a high degree of specialization to the host environment [25]. Differential expression of proteases in various species of dermatophytes has been related to their adaptation to a diverse range of hosts and course of infections [55,56]. Higher expression could induce higher inflammatory reactions and immune system responses, while lower expression might lead to a more subtle and chronic infection [55,56].

*Trichosporon* species seem to maintain their fitness in a wider range of environments than other skin pathogens and may possibly be less specialized. In this sense, it was suggested that the high level of protease expression in *Trichosporon* might indicate that these species are still in the process of adaptation to the animal hosts [55,56]. Accordingly, there are prominent differences in genome size and predicted encoding genes between *Trichosporon* spp. and *Malassezia*. However, further research on *Trichosporon*–host interactions is required to prove such hypotheses.

## What Can Molecular Biology Do to Improve Our Knowledge about *Trichosporon* Infections?

Exciting times are coming in the study of *Trichosporon*, with the development of biological tools that will facilitate the enhancement of our understanding of *Trichosporon* pathogenicity determinants. Genome and RNA sequencing projects will contribute considerably to the understanding of which genes are shared between *Trichosporon* species and/or isolates with differing virulence profiles and which genes are expressed under different pathogenic conditions. Diagnostics, epidemiology, and drug-resistance studies can take dramatic advantage of the genome sequencing of multiple clinical isolates. Protocols for genetic transformation and genetic markers have already been developed for *T*. *cutaneum*, and it should become relatively straightforward to transfer this technology to other *Trichosporon* species. The construction of deletion strains will allow us to interrogate about the participation of several genes as virulence determinants and the role played by the different morphological phases of this fungus in the infection process. In combination with the already established murine and *Galleria mellonella* model systems [19,57], this will permit the use of forward and reverse genetics to interrogate the determinants of pathogenicity, in turn facilitating the development of novel drugs to combat this important fungal pathogen.

## Supporting Information

S1 Table *Trichosporon* species with year of description, MycobankID, and GenBank accession. (XLSX)
